# Evoked Emotions Predict Food Choice

**DOI:** 10.1371/journal.pone.0115388

**Published:** 2014-12-18

**Authors:** Jelle R. Dalenberg, Swetlana Gutjar, Gert J. ter Horst, Kees de Graaf, Remco J. Renken, Gerry Jager

**Affiliations:** 1 Top Institute Food and Nutrition, Wageningen, the Netherlands; 2 Neuroimaging Center Groningen, University Medical Center Groningen, Groningen, The Netherlands; 3 Division of Human Nutrition, Wageningen University, Wageningen, The Netherlands; Saarland University, Germany

## Abstract

In the current study we show that non-verbal food-evoked emotion scores significantly improve food choice prediction over merely liking scores. Previous research has shown that liking measures correlate with choice. However, liking is no strong predictor for food choice in real life environments. Therefore, the focus within recent studies shifted towards using emotion-profiling methods that successfully can discriminate between products that are equally liked. However, it is unclear how well scores from emotion-profiling methods predict actual food choice and/or consumption. To test this, we proposed to decompose emotion scores into valence and arousal scores using Principal Component Analysis (PCA) and apply Multinomial Logit Models (MLM) to estimate food choice using liking, valence, and arousal as possible predictors. For this analysis, we used an existing data set comprised of liking and food-evoked emotions scores from 123 participants, who rated 7 unlabeled breakfast drinks. Liking scores were measured using a 100-mm visual analogue scale, while food-evoked emotions were measured using 2 existing emotion-profiling methods: a verbal and a non-verbal method (EsSense Profile and PrEmo, respectively). After 7 days, participants were asked to choose 1 breakfast drink from the experiment to consume during breakfast in a simulated restaurant environment. Cross validation showed that we were able to correctly predict individualized food choice (1 out of 7 products) for over 50% of the participants. This number increased to nearly 80% when looking at the top 2 candidates. Model comparisons showed that evoked emotions better predict food choice than perceived liking alone. However, the strongest predictive strength was achieved by the combination of evoked emotions and liking. Furthermore we showed that non-verbal food-evoked emotion scores more accurately predict food choice than verbal food-evoked emotions scores.

## Introduction

Consumers show high variability in food choice behavior. The motivators influencing this type of choice behavior are complex and include psychological, physiological, situational, socio-cultural, and intrinsic & extrinsic product characteristics [1]. Despite the complexity of this behavior, choosing what to eat or what to drink is often governed by a fast intuitive process rather than by relatively slow process that involves reasoning [1], [2]. Within this two-system view, intuition is defined as a fast, automatic, effortless, associative, implicit, and an emotionally charged process that is often controlled by habit. Reasoning, on the other hand, involves a slower, serial, effortful, flexible, and a more likely consciously controlled and monitored process [2], [3].

One characteristic of intuition is its emotional basis [2]–[4]. Slovic et al. [3] argued that although rational analysis is important in some situations, reliance on an intuitive system that includes fast emotional processing is more efficient for survival than reasoning. Given the strong relation between nutrition and survival, it is not surprising that emotional valence, measured in liking, was found to be strongly related to experimentally controlled food choice behavior [5], [6]. However, the relation between food choice and liking seems to be weaker in real world situations [5]. This is exemplified by chocolate consumption; although chocolate is highly liked, actual consumption of chocolate varies between consumers and heavily depends on many more factors than merely liking. Therefore, the field of sensory, consumer and food science shifted its focus towards identifying additional motivators for food choice behavior and how these motivators interact with liking.

In recent years, considerable attention is given to food-evoked emotions as motivators for food choice, leading to the introduction of several emotion measurement instruments and guides for measuring food-evoked emotions [1], [7]–[12]. To our best knowledge, the majority of these instruments within the field of sensory, consumer and food science are verbal; participants are instructed to rate emotions that are presented either as single terms or as questions. One popular verbal method is EsSense Profile [13]. EsSense Profile allows for measuring 39 emotions via self-reported intensity scores on a 5-point scale ranging from 0 (not at all) to 4 (extremely). EsSense Profile contains mainly positive emotion terms, since studies have indicated that consumers use (mildly) positive rather than negative emotions when describing their food experiences, a phenomenon called “hedonic asymmetry” [10], [11], [13].

A verbal instrument involves translation of the ‘emotional lexicon’ across cultures and languages, which may complicate interpretation as well as comparisons across studies [14]–[17]. Furthermore, the intuitive nature of emotions would advocate for a more implicit type of measurement [1], [14]. A non-verbal emotion measurement instrument may address these problems. One such instrument is the Product Emotion Measurement Instrument (PrEmo) [14]. PrEmo is an emotion measurement instrument that measures the self-reported intensity of 12 emotions on a 5-point scale similar to EsSense Profile. However, PrEmo presents animated cartoon characters that express emotions instead of presenting emotions verbally. Despite its advantages over a verbal method, PrEmo has been criticized for its low number of (positive) emotions and for not being tailored to food-evoked emotions specifically [13]. Therefore PrEmo may lead to less sensitivity to distinguish between food products.

Most emotion theorists agree that emotions contain at least two qualities: valence and arousal. These can be mapped in an affective space comprised of two orthogonal axes, ranging from unpleasant to pleasant and from calm to excitement, for valence and arousal, respectively [18]–[21]. Not surprisingly, data from food-evoked emotion studies also decompose in these latent variables indicating that emotion measurement provides information on valence as well as arousal for each food product [7], [22]. Previous studies also indicated (sometimes indirectly) a strong relationship between valence and liking. Indeed, many emotion scores correlate moderately to highly with liking scores [7], [22], [23].

However, little is known about whether food-specific emotional profiles contain additional information over liking, in explaining or predicting subsequent food choice behaviour. To our best knowledge most studies on food-related emotions have included attitudinal measures (preference or liking ratings) as an index of the consumer's satisfaction and/or as an estimate of food choice or consumption behavior. However, the ultimate behavior of interest should not be expressed intention but actual choice and/or consumption. Furthermore, previous studies have mostly focused on distinguishing between products based on group averaged emotions scores and not on predicting individualized food choice behavior. These two issues are addressed in the current study by focusing on predicting individualized choice-behavior of the consumer based on liking and emotion measurements.

We hypothesized that product valence and product arousal, in addition to product liking, better predict individualized product choice than merely product liking. To test this hypothesis we used a data set of an experiment in which consumers were invited to rate food products using EsSense Profile, PrEmo, and a VAS liking scale. With a 1-week delay, consumers were re-invited and instructed to choose one product for consumption in a simulated cafeteria setting created in the Restaurant of the Future (RotF), situated in Wageningen, The Netherlands. The RotF is a field laboratory that allows studying food choice behavior in a simulated out-of-home eating and drinking setting.

To elucidate the association with choice, we used product liking, product valence, and product arousal, as predictors in Multinomial Logit Models (MLM) [24]–[26]. These models allow estimation of choice between multiple alternatives. Possible predictors can include variables associated with the choice alternatives (e.g. emotion scores, liking or test setting) and individual-specific variables (e.g. gender or age).

## Methods

The data we used is part of an ongoing series of studies conducted at the Wageningen University (acquired by S.G.). Additional results from these studies will be reported elsewhere (see e.g. [27]).

### Participants

One hundred twenty-three healthy, Dutch speaking, participants (90 women) were recruited from Wageningen and surrounding areas. Inclusion criteria were: previous experience with the product category (defined as at least being incidental consumers of breakfast drinks), aged 18 – 55 years, and normal weight (BMI 18.5 – 27 kg/m^2^). Exclusion criteria were: a change of body weight of more than 5 kg during the last two months, having food allergy or food intolerance, and, for women, being pregnant or lactating. Table 1 shows participant characteristics, including a categorization of breakfast drink consumption in incidental, regular and frequent consumers.

**Table 1:** Participant characteristics.

	Incidental users (N = 27)		Regular users (N = 68)		Frequent users (N = 28)	
	Criterion: 1–14 ever in lifetime		Criterion: 1 – 9 times a year		Criterion: 10 or more times a year	
Gender	Female	Male	Female	Male	Female	Male
N	23	4	47	21	20	8
Age (y)	26.13 (9.44)	32.25 (9.54)	25.85 (11.51)	26.14 (10.22)	25.1 (8.77)	29.38 (12.52)
BMI (kg/m2)	22.35 (2.16)	23.38 (1.36)	21.63 (2.02)	22.24 (1.95)	21.85 (1.84)	21.62 (1.83)

Participant characteristics (means± sd) and classification in incidental, medium, and frequent users of breakfast drinks. Non-users (criterion: never in lifetime) were excluded from the study.

Participants were ignorant to the exact aim of the study and were informed that the researchers were interested in product evaluation differences between consumers and non-consumers of breakfast drinks.

Participants received financial compensation for participation and completed a consent form. Furthermore, the Medical Ethical Committee of Wageningen University gave ethical approval for the study.

### Products

The products used in this study were breakfast drinks. These drinks were commercially available at the time of the study. Table 2 shows an overview of the breakfast drinks, including information on brand, flavor and a short description of the sensory attributes. In the remainder of this paper, the breakfast drinks will be referred to as product A – G (see Table 2).

**Table 2:** Product information for the seven test products.

Product ID	Product brand	Flavor	Short product description
A	Campina, “Good morning” 1	Orange, mango and banana	Dairy based, liquid breakfast drink with grains
B	Hero, “Fruit Breakfast” 2	Forest fruit	Juicy based liquid breakfast drink with grains
C	Hero, “Fruit Breakfast” 2	Orange and banana	Juicy based liquid breakfast drink with grains
D	Campina, “Good morning” 1	Peach and apricot	Dairy based liquid breakfast drink with grains
E	Campina, “Good morning” 1	Strawberry, kiwi and banana	Dairy based liquid breakfast drink with grains
F	Friesche Vlag, “Breaker”	Strawberry and banana	Dairy based semi-liquid (yoghurt like) breakfast drink, no grains
G	Friesche Vlag, “Breaker”	Peach	Dairy based semi-liquid (yoghurt like) breakfast drink, no grains

1 Translated from the Dutch product brand name Campina, “Goede Morgen”.

2 Translated from the Dutch product brand name Hero, “Fruit Ontbijt”.

The table shows the product information of all products that were used in the study.

As our primary interest in this study was on intrinsic product properties and the emotions they evoke, the breakfast drinks were presented unbranded (without brand or packaging information) during all measurements of this study.

### Design & Procedure

Participants took part in two test sessions with an interval of one week. Testing took place in the morning, either at 7:30 am or at 9:30 am. Participants were scheduled at the same time slot for both sessions and were not allowed to eat two hours before the start of each test session.

### Session 1

At the start of session 1, participants were seated in secluded sensory testing booths and were given written instructions, describing the experiment.

The first test session consisted of two blocks, separated by a 10-minute break to minimize fatigue. During block 1, participants evaluated product-evoked emotions using the emotion profiling method PrEmo (∼25 minutes), whereas participants evaluated product-evoked emotions using EsSense Profile (∼35 minutes) in block 2.

Both blocks were divided in seven randomized trials (one per product). During every trial, a test sample of a breakfast drink (15 ml) was served in a transparent cup (refrigerated at 4°C until the moment of serving) together with a teaspoon. Participants were instructed to first stir the breakfast drink, and then to taste a spoonful of the drink. Subsequently, the participant was tasked to score his or her evoked emotions using the emotional profiling method. Following emotion profiling during block 2, participants were instructed to taste the current breakfast drink once more and rate its overall liking on a 100-mm visual analogue scale, anchored “dislike extremely” and “like extremely”. The trials were separated by a one-minute break, in which participants had to clean their palate and rinse their mouth with water and unsalted crackers.

### Session 2

During session 2, actual food choice was measured. Participants were instructed to come to the RotF and were seated at two large tables joining other participants. They were then presented with seven samples of the breakfast drinks served in transparent cups as described in session 1, which were placed in a randomized order on a tray. Participants were instructed to taste all seven unlabeled breakfast drinks and to point out which one they preferred to have for breakfast (no other breakfast products were served).

### Statistical analysis

All analyses were performed in R (www.r-project.org, version 3.0.2, 2013-09-25).

First, the emotion scores of all participant-product combinations (861, i.e. 7 products for 123 participants) were concatenated to form an 861 × 12 matrix **P** and an 861 × 39 matrix **E** for the PrEmo and EsSense Profile data, respectively. Subsequently, the mean score per emotion was removed per participant, eliminating possible offset-biases between participants. In other words, the average emotion response was removed per participant, such that the focus is on within-participant variability across products.

To form a succinct representation of the data, the demeaned matrices **P** and **E** were decomposed into principal components (PCs) by using singular value decomposition. The scores on the first two PCs were used for further analysis. To provide insight in these PCs for both data sets, we will show a biplot of the components and indicate their associated explained variance.

Multinomial logit models (MLMs) were used to predict product choice. MLMs are provided in package mlogit (version 0.2–4). For the current study, we used two PC scores from the emotion data as well as perceived liking ratings. Because PC-scores and liking ratings are expressed in different units, we centered and scaled these data to a standard deviation of 1, such that their beta estimates within the MLMs can be compared. Because we were interested in the predictive value of the independent variables on product choice as well as finding an optimal model, we constructed a total of 7 statistical models (see Table 3). These models contained different combinations of the independent variables and were compared in terms of model performance. Performance was assessed using likelihood ratio tests between the model fits. These tests allow evaluating whether addition or replacement of independent variables significantly increases the goodness of fit. Comparisons were executed in a stepwise forward selection procedure: a procedure in which models of increasing complexity are evaluated in each step. When model comparisons are made, we will report the associated χ^2^ statistic. For the optimal model, we will report the corresponding beta estimates. Furthermore, MLMs rely on the independence of irrelevant alternatives (IIA) hypothesis (i.e., the assumption that choice estimation on one alternative is independent from the other alternatives). This hypothesis was tested with the Hausman-McFadden Test [28], which evaluates the degree of change in parameter estimates in the model when one choice alternative is removed from the data, compared to the original model.

**Table 3:** Constucted Multinomial logit models.

Model ID	Dependent Variable	Independent variable(s)
1	Choice	Liking
2		PrEmo PC1
3		EsSense PC1
4		Liking, PrEmo PC1
5		Liking, EsSense PC1
6		Liking, PrEmo PC1, PrEmo PC2
7		Liking, EsSense PC1, EsSense PC2

This table shows all MLMs that were constructed along with their identifier (model ID).

**Figure 1:**
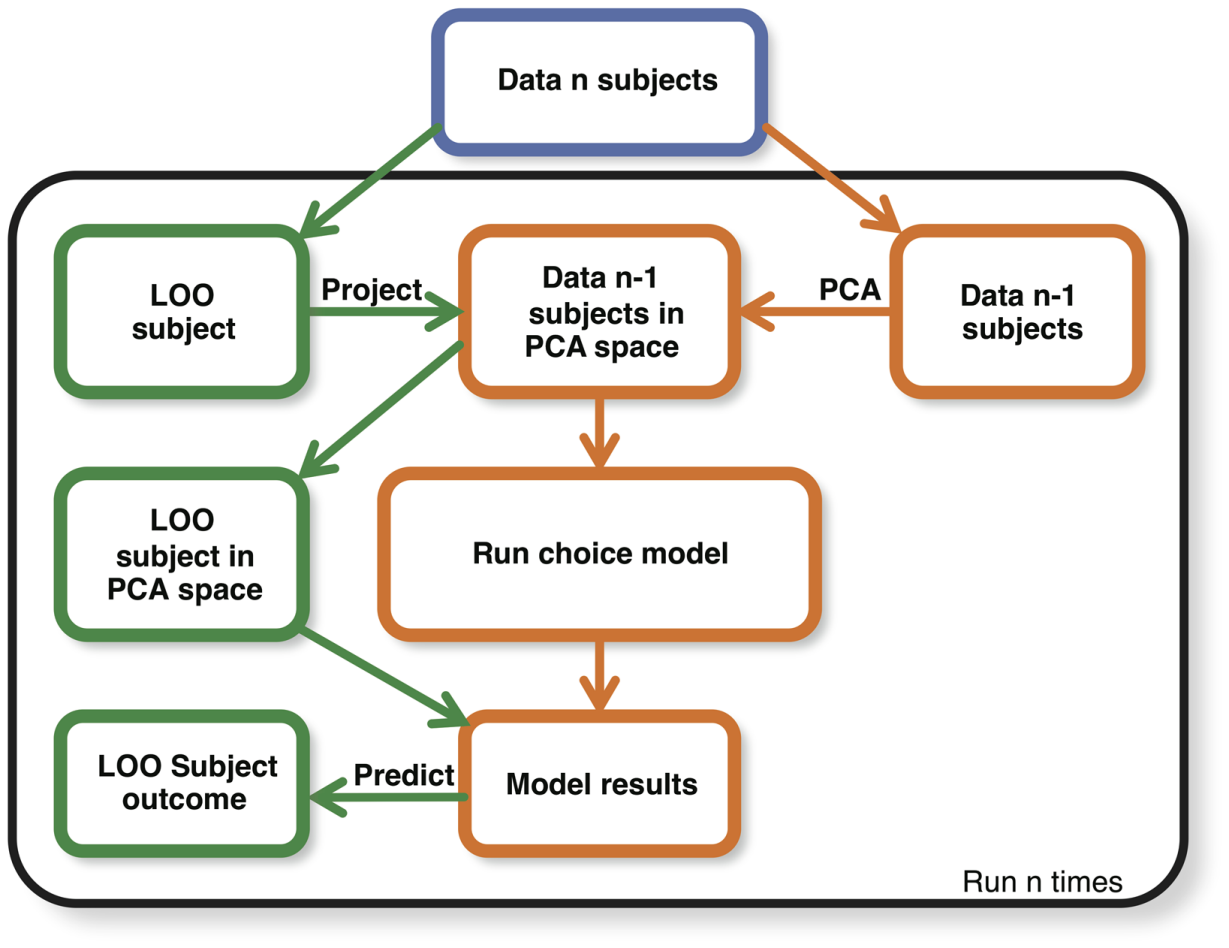
Schematic overview of the Leave One Out Cross Validation (LOOCV). This schematic overview shows how the LOOCV was implemented for unbiased prediction of individualized product choices. The complete data set is indicated in blue. Data operations with the independent individual are given in green, while data operations with the data from all remaining individuals are given in orange. The data operations within the plane that is bordered in black were repeated for every individual.

The quality of fit for the MLMs was assessed in 2 ways. First, we indicated the effect size by reporting McFadden's adjusted r^2^. Note that McFadden indicated that r^2^ values between 0.2 to 0.4 represent an excellent fit [29]. Furthermore, we regarded the MLM as a machine-learning algorithm and evaluated its predictive value by leave-one-out cross-validation (LOOCV). LOOCV allows for an unbiased prediction estimate, because the choice of every single individual is predicted independently from all other individuals. Fig. 1 shows a schematic overview of the LOOCV process. For every cycle in the LOOCV procedure, data from a single subject was isolated first; the so-called left one out (LOO) subject. Subsequently the emotion data of n-1 subjects was decomposed into PCs via a principal component analysis (PCA), independently from the data of the LOO subject. The resulting rotation matrix of this PCA was used to perform the same PCA rotation on the emotion data of the LOO subject. Next, the MLM was estimated using the PC1 scores and liking as independent variables, and choice as dependent variable. Finally, the estimated parameters of the MLM were used to generate a choice prediction for the LOO subject. Per individual, this prediction provided a choice probability for each of the 7 products. To indicate the predictive value of the optimal model, we converted these choice probabilities to a rank-order per participant, running from 1 (product with highest chance of being chosen) to 7 (product with lowest chance being chosen). To graphically show the result, we will plot the frequency of final product choices per predicted rank and indicate performance on chance level for comparison. Within this graph perfect prediction performance would reflect in all final product choices predicted as rank 1, while the worst performance would reflect all final product choices predicted as rank 7.

Because PCs are recalculated for n-1 participants within each LOOCV-cycle, the emotion loadings on the PCs are prone to (small) deviations. To provide information on this, the emotion loadings on PC1and PC2, along with their deviation are provided in S1 Table of the supplementary materials. Furthermore, we carried out additional analyses to indicate whether the remaining principal components contained any additional predictive strength (see S1 Supporting Information.).

### Data availability

The emotion-measurement datasets are given in S2 Table and S3 Table.

## Results

Table 4 shows the relative popularity of all product alternatives in the experiment. Product F and product G clearly stood out from the rest, as these were chosen most often. As expected this effect was also reflected in the perceived liking associated with these products.

**Table 4:** Product choice and liking details.

Product	A	B	C	D	E	F	G
Choice (%)	5.7	3.3	2.4	3.3	10.6	41.5	32.5
Liking (1–100)	56.5±21.8	42.6±25.3	43.1±26.4	47.0±22.5	55.3±21.0	64.2±23.4	61.9±24.8

The table shows the percent choice of each product and their associated percieved liking (mean ± sd).

**Figure 2:**
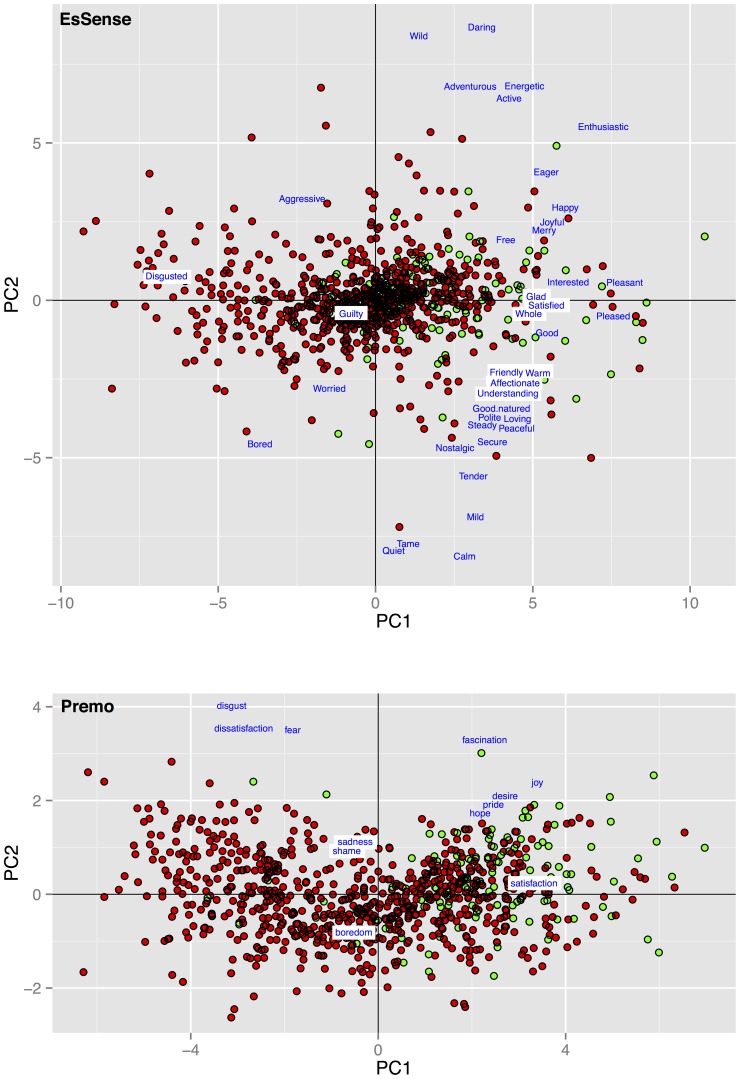
PCA biplots of EsSense and PrEmo emotion data. The figure shows a biplot a PCA performed on the EsSense and PrEmo emotions. Every data point represents all rated emotions per method that were rated by a participant on a single product. The data points are colored based on empirical choice; chosen products are colored green and not chosen products are colored red. In blue we plotted the loadings of all emotion variables.

Fig. 2 provides two biplots of the emotion data in which the first principal component (PC1) is plotted against the second principal component (PC2) for EsSense Profile and PrEmo data, respectively. Within these plots, every data point represents the emotions for a single product scored by a single participant. PC1 reflected emotions ranging from unpleasant to pleasant and explained 41% and 65% of the total variance for EsSense Profile and PrEmo data, respectively. PC2 reflected emotions ranging from tranquil to energetic and explained 9.2% and 7.6% of the total variance for EsSense Profile and PrEmo respectively.

**Figure 3:**
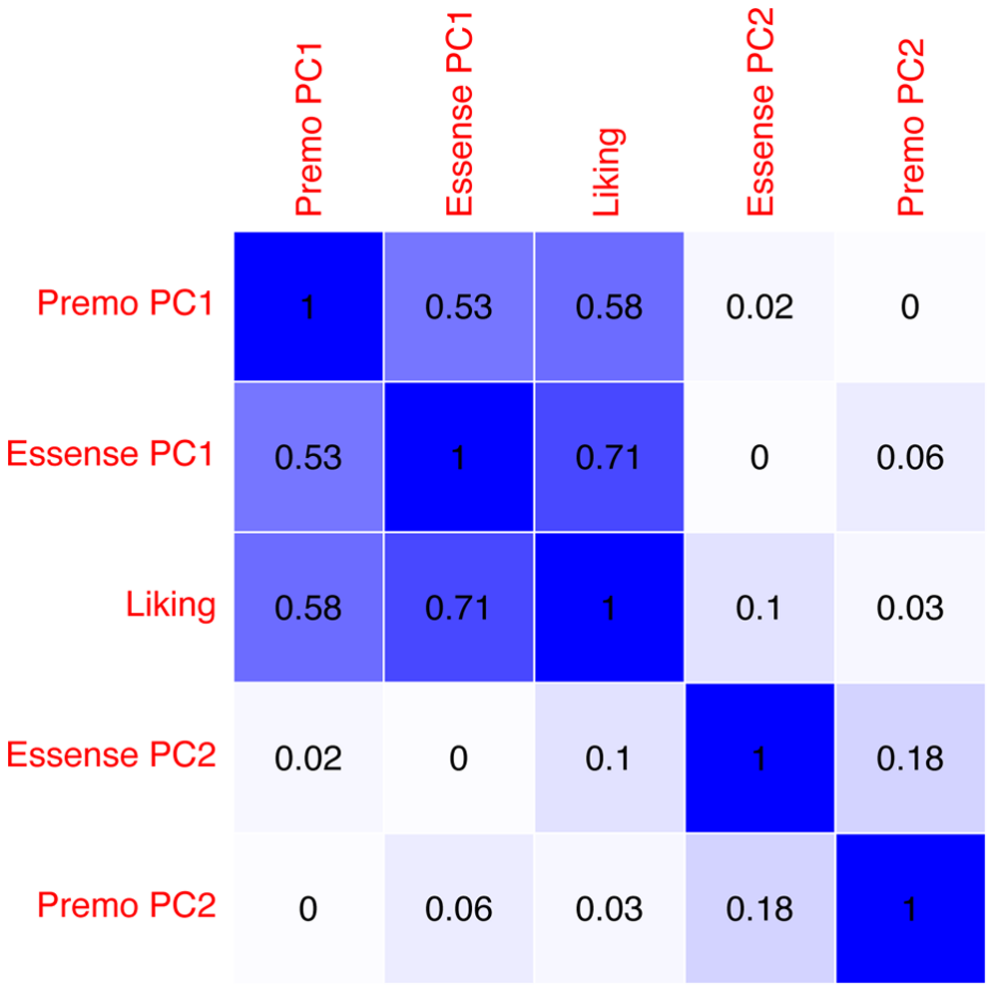
Correlations between the independent variables. The figure shows the Pearson correlations between the independent variables that were used in the analysis. The figure indicates high correlations between liking and the valence components from the emotion measurement methods. Furthermore, there is a weak correlation between the arousal components of the emotion measurement methods.

Fig. 3 shows a matrix indicating the Pearson correlation values between all used independent variables. As expected, Liking, Premo PC1 and EsSense Profile PC1 show high correlations (0.53 ≥ r ≤ 0.71). Furthermore, PC2 of both emotion measurement methods show a weak correlation (r  =  0.18).

To test the hypothesis that 1) measuring emotions has additive predictive value over merely perceived liking for choice and 2) whether a more intuitive method better predicts choice than a verbal method, we estimated the MLM models presented in Table 3 and compared their goodness of fit. First, we compared Model 1 to 2 and 1 to 3. These model comparisons showed that models that contain either PrEmo PC1 or EsSense PC1, significantly better predict product choice than a model containing only liking (Premo: χ^2^  =  12.47, p<0.001, EsSense: χ^2^  =  1.77, p<0.001). However, comparisons between Model 2 and Model 4 as well as Model 3 and Model 5 indicated that the combination between PC1 and perceived liking was favorable over only PC1 (Premo: χ^2^  =  10.07, p<0.005, EsSense: χ^2^  =  4.94, p<0.05). When comparing Model 4 to Model 5, the model containing PrEmo PC1 significantly improved the model fit over EsSense Profile PC1 (χ^2^  =  15.82, p<0.001). Further model comparisons with Model 6 and Model 7, indicated that adding PC2 as extra independent variable, did not significantly improve the model fits (Premo: χ^2^  =  3.6, p = 0.06, EsSense: χ^2^  =  0.60, p = 0.44).

For Model 4, the best fitting model (McFadden adj. r^2^  =  0.202), PrEmo PC1 (β  =  0.78, p <0.001) and perceived liking (β  =  0.55, p <0.005) were both positively associated with product-choice. Testing the IIA hypothesis (i.e. the assumption of independence between choice alternatives) showed that IIA could not be rejected (χ^2^(7)  =  9.57, p = 0.21).

**Figure 4:**
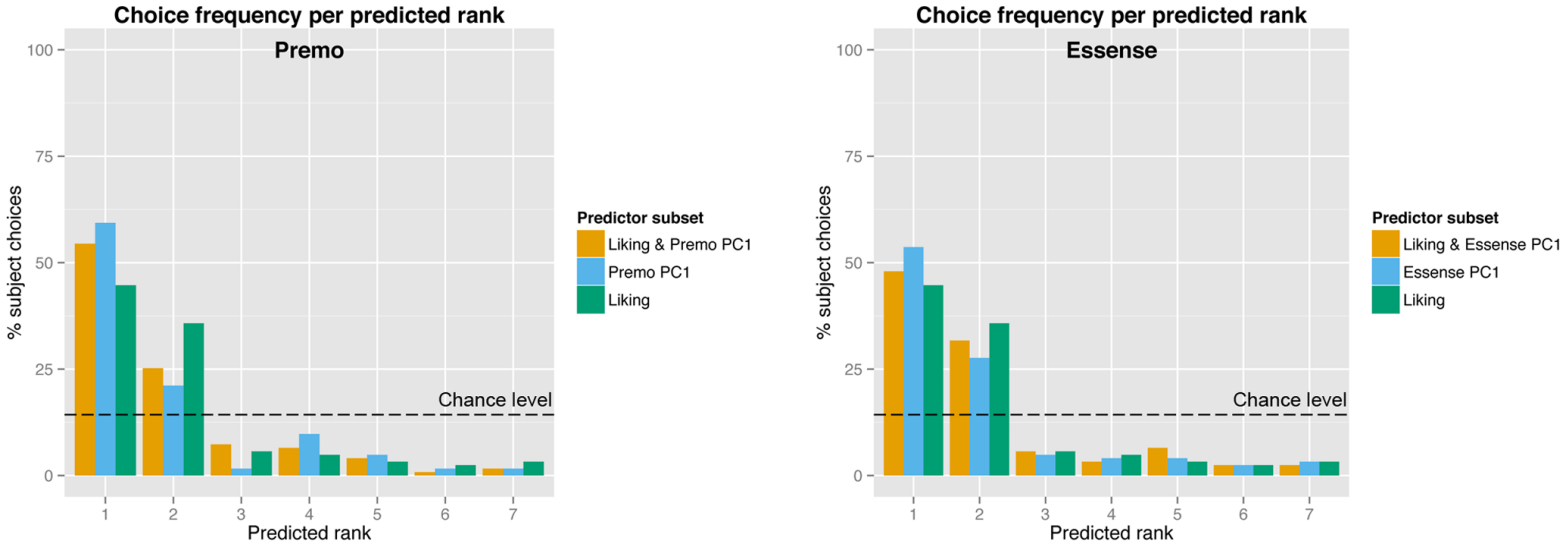
Result of the LOOCV predictions. This figure shows the prediction outcomes of the LOOCV using different subsets of the predictors Liking, Premo PC1 and Essense PC1. For the prediction, the multinomial logit model was used to create a distribution of p-values that represented the chances for every product to be chosen by each individual. These p-values were transformed in ranks; the product with the highest predicted chance of being chosen received rank 1 and the product with the lowest predicted chance of being chosen received rank 7. In the figure we show the percentage of final product choices per predicted rank (e.g. product choice for 54% of the participants, was (correctly) predicted as rank 1 by the model when using Liking & Premo PC1 as predictors). The dashed line indicates how the model would perform on chance level (14.3%). Note that an improvement in prediction performance would reflect a distribution change from right to left in the plot.

To indicate the predictive value of several models, we performed LOOCVs on Model 1 to Model 5. Fig. 4 shows the outcome of the LOOCV predictions. The figure shows the percentage of empirical product choices as a function of predicted rank. As can be seen, the models perform far above chance level (dashed line). The best fitting model (Model 4, containing Liking and Premo PC1 as independent variables) correctly predicted 54.5% of all empirical choices as rank 1 and 25.2% as rank 2.

## Discussion

The aim of this study was to investigate the added predictive value of food-evoked emotions for food-choice in a simulated eating environment. For this purpose, we used a data set in which food-evoked emotions, perceived liking, and 1-week delayed food choice of 7 breakfast drinks were measured. We showed, for the first time, that measuring food-evoked emotions, in addition to merely perceived liking, improves the estimation of individualized food choice. Cross-validation of the results showed that we were able to successfully predict product choice, for over 50% of the individuals. The number of correct predictions rose to nearly 80% when looking at the top 2 product candidates.

### Liking, valence & arousal as predictors for choice

Although previous studies showed that product liking is associated with product choice, measuring merely product liking is an insufficient predictor for product choice. Therefore increasing interest has been given to food-evoked emotions. In previous research it was argued that emotions drive choice behavior, and measuring these emotions allows for differentiation between products [7], [13], [22], [30], [31]. In line with this research we showed that product liking is indeed a significant predictor for 1-week-delayed product choice. However, a model containing merely product liking only moderately fitted the choice behavior (see Fig. 4). To indicate the additive predictive value of evoked emotion measurement we decomposed the emotion data into PCs. As expected, the first two PCs of the emotion scores could be interpreted as product valence and product arousal. Model comparisons showed that product valence was a strong predictor for food choice. Also, adding product valence as an extra predictor in addition to product liking, significantly improved the model fit for choice estimation indicating that both valence and liking contain mutually exclusive information, despite their high correlation (see Fig. 3).

To our surprise, we found no significant relation between arousal and choice on a group level, indicating that valence scores extracted from emotion data provide sufficient information. These results are also illustrated in Fig. 2. The figure shows that chosen products received more positive valence scores. However, chosen products are almost equally distributed over the entire arousal axis. We conclude from this finding that there is large agreement between participants in associating perceived valence and product choice, whereas the associating between perceived arousal and choice appear to be subject to large interindividual differences. This result does not necessarily mean that arousal and choice are unrelated. Not finding a relation on a group level may be explained by previous work on optimal arousal theory. The optimal arousal theory assumes that the stimulus' arousal level is evaluated with respect to the optimal arousal level of the individual at the moment of consumption [32]. In other words: the ideally induced arousal level of e.g. a breakfast drink within a consumer depends on the optimal arousal level of that consumer during breakfast. If the optimum arousal level differs highly between individuals, perceived arousal scores may not or weakly associate with product choice in a group analysis. Here, we did not have information on the optimal arousal level of participants. To improve choice estimation, we, therefore, recommend measuring appropriate personal characteristics in future research.

Our analysis was centered on the first two principal components within both datasets. These components have a clear interpretation. Additional analysis presented in S1 Supporting Information shows that several smaller principal components in the EsSense Profile data set contained additional predictive strength. However, we found these components hard to interpret. Further analysis on these components showed that they are far less stable than PC1 (see S1 Table and S1 Supporting Information).

### Verbal versus Non-verbal measurement of emotions

Within our study we used EsSense Profile as well as PrEmo to measure food evoked-emotions. Whereas EsSense Profile is a verbal emotion measurement instrument, PrEmo presents emotions non-verbally as animated cartoon characters. Although both methods perform well on discriminating products, it remained unclear how both methods performed as predictor for product choice.

PCAs showed that the first two PCs in EsSense Profile data capture far less variance than in PrEmo. This difference indicates that a larger proportion of the variance within the PrEmo dataset captures valence information compared to EsSense Profile. This could be explained by the dichotomous distribution of emotions in PrEmo; the instrument measures 6 positive and 6 negative emotions, while EsSense Profile is very imbalanced with 25 positive, 3 negative and 11 uncategorized emotions [13]. Model comparisons showed that product valence measured by PrEmo (PrEmo PC1), as well as product valence measured by EsSense Profile data (EsSense PC1) improve choice estimation. However, a direct comparison between PrEmo and EsSense Profile showed that data measured by PrEmo better predicts product choice.

A possible reason for this result is that emotional content in non-verbally expressed emotions is processed more intuitively and, therefore, more closely resembles intuitively experienced emotions. Evidence for this hypothesis stems from EEG-experiments showing that emotion processing is faster for facial expressions than for emotional words [33]–[35].

Furthermore, the average liking scores for each product fluctuated around neutral (50% of the scale), while the standard deviations ranged between 20 and 25% of the liking scale. This result indicates that there is a considerable amount of dislikers for each product. This may sound counterintuitive as the sample included only consumers of breakfast drinks. However, being familiar with and a user of the product category, does not imply that each product is equally well liked. Because the range of emotions in PrEmo is more dichotomously distributed, the instrument may allow product dislikers to express their disliking more accurately, while EsSense Profile is “*aimed at product users who typically like the product*” [13] and may, therefore, capture disliking less accurately.

A further limitation in the comparison between both methods is that all participants first completed PrEmo followed by EsSense Profile within the experiment. The fixed order of tests was based on the intuitive nature of PrEmo. According to Desmet et al. (2000) “*asking participants to describe their emotional response will require cognitive involvement, which may influence the measurement*”. Therefore, running EsSense Profile ratings prior to PrEmo, would induce priming of the emotional lexicon within the participants. However, we cannot exclude the possibility of experiment fatigue experienced by the participants, leading to less reliable scores on the EsSense Profile method.

### Future work

Here, we focused on evoked-emotions based on blind product evaluation (i.e. products were presented without brand or package information). In contrast, consumers are provided with much more information when making decisions about product choice and product consumption in a real world setting. Therefore, we need to investigate how packaging affect food-evoked emotions and whether potential differences in evoked emotions alter the relation between evoked emotions and product choice. Furthermore, more information is needed on personal characteristics such as attitudes to food as well as optimal evoked arousal levels to improved food choice estimation.

### Conclusion

In the current study, we showed that we were able to indicate the relation between evoked emotions and food choice using a combination of existing methods. MLMs showed that evoked emotions better predict food choice than perceived liking alone. However, the combination of emotion- and liking measures had the strongest predictive value for product choice. With cross-validation we showed that we were able to predict individualized choice with high accuracy. Furthermore we showed that measurement of non-verbal food-evoked emotions more accurately predict product choice than verbal food-evoked emotions.

## Supporting Information

S1 Table **PC1 variable loadings for EsSense Profile and PrEmo questionaires.** The table shows the PC1 variable loadings for the emotion variables that were measured with the EsSense Profile and PrEmo questionaires. Furthermore, the table shows the leave-one-out standard deviations (SD) that were calculated over the LOOCV cycles. The low SD values indicate that the variable loadings remained very stable.
(DOCX)

S2 Table **Data from EsSense Profile.** The data set contains subject ID, product ID, liking scores (100 point VAS), 1-week delayed choice score (1  =  chosen, 0  =  not chosen), and all scores on the emotions from the EsSense Profile method.
(TXT)

S3 Table **Data from PrEmo.** The data set contains subject ID, product ID, liking scores (100 point VAS) and all scores on the emotions from the PrEmo method.
(TXT)

S1 Supporting Information **Additional analysis on predictive value of all Principal Components.** Additional analysis investigating the predictive value of the remaining principal components for both data sets.
(DOCX)
