# Supplementary material for: Evoked Emotions Predict Food Choice
Source: PLoS One. 2014 Dec 18;9(12):e115388. doi: 10.1371/journal.pone.0115388 (PMC4270769; doi:10.1371/journal.pone.0115388)
Supplement: S1 Table — PC1 variable loadings for EsSense Profile and PrEmo questionaires. The table shows the PC1 variable loadings for the emotion variables that were measured with the EsSense Profile and PrEmo questionaires. Furthermore, the table shows the leave-one-out standard deviations (SD) that were calculated over the LOOCV cycles. The low SD values indicate that the variable loadings remained very stable. (DOCX) [file pone.0115388.s001.docx]

**Table S1. PC1 variable loadings for EsSense and PrEmo questionaires.**

| **EsSense** | | | | | | | | |
| --- | --- | --- | --- | --- | --- | --- | --- | --- |
| **Emotion** | *Pleasant* | *Active* | *Aggressive* | *Adven-turous* | *Under-standing* | *Polite* | *Happy* | *Worried* |
| **Score^1^** | 0.279 | 0.150 | -0.082 | 0.106 | 0.148 | 0.128 | 0.218 | -0.052 |
|  |  |  |  |  |  |  |  |  |
| **Emotion** | Whole | Energetic | Enthusiastic | Interested | Glad | Good | Good natured | Eager |
| **Score** | 0.172 | 0.167 | 0.255 | 0.216 | 0.180 | 0.192 | 0.141 | 0.191 |
|  |  |  |  |  |  |  |  |  |
| **Emotion** | Affectionate | Calm | Loving | Mild | Nostalgic | Guilty | Steady | Quiet |
| **Score** | 0.156 | 0.100 | 0.140 | 0.112 | 0.089 | -0.027 | 0.121 | 0.020 |
|  |  |  |  |  |  |  |  |  |
| **Emotion** | Tame | Tender | Pleased | Daring | Secure | Bored | Satisfied | Disgusted |
| **Score** | 0.037 | 0.110 | 0.268 | 0.119 | 0.131 | -0.130 | 0.195 | -0.234 |
|  |  |  |  |  |  |  |  |  |
| **Emotion** | Peaceful | Joyful | Friendly | Free | Merry | Warm | Wild |  |
| **Score** | 0.153 | 0.198 | 0.166 | 0.146 | 0.189 | 0.178 | 0.049 |  |
| **Premo** | | | | | | | | |
| **Emotion** | desire | satisfaction | pride | hope | joy | fascination | disgust | dis-satisfaction |
| **Score** | 0.325 | 0.404 | 0.299 | 0.260 | 0.412 | 0.276 | -0.379 | -0.348 |
|  |  |  |  |  |  |  |  |  |
| **Emotion** | shame | fear | sadness | boredom |  |  |  |  |
| **Score** | -0.081 | -0.221 | -0.064 | -0.063 |  |  |  |  |

**^1^** The scores are the calculated mean over all 123 LOOCV cycles. The Standard Deviation (SD) was also calculated. For all emotions the SD ranged between 0.001 and 0.002.

The table shows the PC1 variable loadings for the emotion variables that were measured with the EsSense Profile and PrEmo questionaires. Furthermore, the table shows the leave-one-out standard deviations (SD) that were calculated over the LOOCV cycles. The low SD values indicate that the variable loadings remained very stable. Note that these estimators are equivalent to jackknife estimators from the jackknife resampling method.
