# Supplementary material for: Evoked Emotions Predict Food Choice
Source: PLoS One. 2014 Dec 18;9(12):e115388. doi: 10.1371/journal.pone.0115388 (PMC4270769; doi:10.1371/journal.pone.0115388)
Supplement: S1 Supporting Information — Additional analysis on predictive value of all Principal Components. Additional analysis investigating the predictive value of the remaining principal components for both data sets. (DOCX) [file pone.0115388.s004.docx]

**Supporting Information S1**

**Title: Additional analysis on predictive value of all Principal Components.**

In the manuscript we carried out an analysis using the first two principal components (PCs) of the EsSense Profile and the PrEmo data sets, because they have a clear interpretation. However, the remaining components may contain predictive power. Therefore, we carried out additional model comparisons, using stepwise forward regression, to indicate whether addition principal components contained predictive value for final choice prediction. The results indicated that EsSense Profile PC19 and PC29 contained additional predictive power, whereas we found no extra predictive power for the remaining PCs within the PrEmo data set. Although PC19 (1.3% variance explained) and PC29 (0.9% variance explained) improved the EsSense Profile model for predicting final choice, a direct comparison between the updated EsSense Profile model and the PrEmo model still indicated that PrEmo showed greater predictive strength. The model comparisons are denoted in Table SI 1.1.

| **Table SI 1.1: EsSense Profile model comparisons** | | | | | |
| --- | --- | --- | --- | --- | --- |
| **Constructed models** | **McFadden R^2^** | **Model comparison statistics** | | | |
|  |  | **Log-Likelihood** | **Df** | **χ^2^** | **p-value** |
| Liking | 0.14 | -153.43 | - | - | - |
| Liking + Essense PC1 | 0.16 | -150.05 | 1 | 6.76 | < .01 |
| Liking + Essense PC1 + PC19 | 0.17 | -148.01 | 1 | 4.09 | < .05 |
| Liking + Essense PC1 + PC19 + PC29 | 0.18 | -144.95 | 1 | 6.11 | < .05 |
| Liking + Premo PC1 | 0.20 | -141.88 | -2 | 6.15 | < .05 |

The table shows additional model comparisons for the EsSense Profile dataset.

Each model was compared to the previous model using likelihood ratio tests.

Furthermore, we investigated the loadings as well as the stability of the loadings within the leave-one-out cross-validation (LOOCV) mentioned in the manuscript. The results are depicted in Table SI 1.2. Although these components were related to final choice, the table shows that these components are unstable, especially compared to PC1 (see Table S1). Furthermore, the high LOOCV standard deviations of the loadings indicate that the components are fairly unstable, making EsSense Profile PC19 and PC29 less likely to contain predictive value in new data sets.

| **Table SI 1.2: EsSense Profile PC19 & PC29 loadings** | | | | |
| --- | --- | --- | --- | --- |
| **Emotion** | **PC19 mean LOOCV loadings** | **PC19  LOOCV SD** | **PC29 mean LOOCV loadings** | **PC29  LOOCV SD** |
| Pleasant | -0.06 | 0.07 | 0.12 | 0.16 |
| Active | -0.19 | 0.09 | 0.08 | 0.11 |
| Aggressive | -0.05 | 0.04 | -0.03 | 0.09 |
| Adventurous | -0.12 | 0.06 | -0.12 | 0.07 |
| Understanding | 0.05 | 0.09 | 0.03 | 0.09 |
| Polite | -0.06 | 0.09 | -0.04 | 0.08 |
| Happy | 0.00 | 0.04 | -0.04 | 0.12 |
| Worried | 0.03 | 0.10 | -0.03 | 0.05 |
| Whole | **0.27** | 0.12 | -0.10 | 0.05 |
| Energetic | -0.07 | 0.05 | -0.12 | 0.06 |
| Enthusiastic | 0.06 | 0.06 | -0.05 | 0.13 |
| Interested | -0.12 | 0.04 | 0.00 | 0.10 |
| Glad | 0.19 | 0.05 | 0.15 | 0.14 |
| Good | 0.02 | 0.06 | 0.12 | 0.14 |
| Good.natured | 0.06 | 0.10 | -0.05 | 0.11 |
| Eager | -0.16 | 0.06 | -0.10 | 0.05 |
| Affectionate | 0.05 | 0.07 | 0.04 | 0.12 |
| Calm | -0.13 | 0.07 | -0.03 | 0.06 |
| Loving | 0.12 | 0.07 | -0.08 | 0.11 |
| Mild | -0.02 | 0.09 | 0.06 | 0.08 |
| Nostalgic | 0.15 | 0.07 | 0.05 | 0.08 |
| Guilty | 0.08 | 0.08 | -0.09 | 0.10 |
| Steady | **0.32** | 0.15 | -0.04 | 0.08 |
| Quiet | **0.24** | 0.08 | -0.03 | 0.04 |
| Tame | -0.05 | 0.08 | 0.01 | 0.06 |
| Tender | **-0.25** | 0.14 | -0.01 | 0.11 |
| Pleased | -0.05 | 0.09 | **-0.21** | 0.08 |
| Daring | 0.17 | 0.08 | 0.11 | 0.11 |
| Secure | -0.02 | 0.09 | 0.08 | 0.10 |
| Bored | 0.00 | 0.03 | -0.02 | 0.06 |
| Satisfied | 0.14 | 0.06 | 0.01 | 0.09 |
| Disgusted | -0.20 | 0.05 | 0.18 | 0.07 |
| Peaceful | **-0.24** | 0.07 | **0.32** | 0.30 |
| Joyful | -0.07 | 0.07 | 0.11 | 0.17 |
| Friendly | -0.17 | 0.06 | -0.13 | 0.12 |
| Free | -0.06 | 0.08 | -0.10 | 0.09 |
| Merry | 0.00 | 0.05 | -0.17 | 0.07 |
| Warm | -0.11 | 0.10 | **-0.35** | 0.16 |
| Wild | **0.23** | 0.08 | 0.12 | 0.10 |

The table shows the mean loadings and associated standard deviations produced by leave-one-out cross-validation. The mean loadings greater than .2 or lower than -.2 have been depicted in bold. Note that these estimators are equivalent to jackknife estimators from the jackknife resampling method.
